# The psychometric properties of the WISC-IV verbal comprehension and working memory indices among visually impaired students in Sudan

**DOI:** 10.3389/fpsyg.2025.1451418

**Published:** 2025-08-18

**Authors:** Mohamed Abdalazeem Alhaj Salih, Abeer Abdelrahman Khalil, Salaheldin Farah Attallah Bakhiet

**Affiliations:** ^1^Department of Psychology, University of Khartoum, Khartoum, Sudan; ^2^Gifted Education Program, Department of Special Education, College of Education, Administrative and Technical Sciences, Arabian Gulf University, Manama, Bahrain

**Keywords:** WISC-IV, verbal comprehension, working memory, visually impaired students, Khartoum, Sudan

## Abstract

**Background:**

Intelligence testing in Sudan began in 1946, with efforts to adapt and validate international assessments starting in 1964. However, no intelligence test had been specifically tailored for students with visual impairments.

**Aims:**

This study aimed to fill this gap by adapting the Verbal Comprehension and Working Memory indices of the WISC-IV for use with visually impaired children and assessing their reliability and validity.

**Methods:**

The study involved 166 visually impaired students (57.83% male, 42.17% female), aged 6–16 years, drawn from schools in Khartoum.

**Results:**

The adapted indices demonstrated strong reliability and validity, supporting their suitability for use with Sudanese students who have visual impairments.

**Conclusion:**

The findings support the use of the adapted Verbal Comprehension and Working Memory indices to assess verbal intelligence in visually impaired students in Sudan for both diagnostic and evaluative purposes.

## Introduction

Psychological research highlights the importance of designing intelligence assessments for individuals with disabilities, such as visual impairment that do not affect cognition (Allam, 2000). Cassar and Lucchese (2016) noted that these assessments measuring mental performance are necessary for diagnosis, guidance, and rehabilitation. However, the measurement of intelligence remains a controversial topic in psychology due to concerns about biases and discriminatory structures that have been integrated into assessments developed for quantifying intelligence, Bahadır and Bahadır (2024) stress it’s essential to focus on whether all individuals residing in that country benefit equally and according to their needs from the services provided. We believe that one of the most important of these services is intelligence tests for people with disabilities. The Wechsler Intelligence Scale for Children (WISC Test), originally developed by David Wechsler in 1949 and currently in its fifth iteration, is one of the most important assessment tools for measuring intelligence. In Sudan, efforts to standardize the WISC have largely focused on the WISC-III (Al-Hussein, 2005, 2008), with the later versions yet to be adapted. However, these measures were developed for sighted individuals, posing challenges in accurately assessing the intelligence of visually impaired students without adaptation.

Globally, 2.2 billion people experience some form of visual impairment, with nearly half in the moderate to severe range (World Health Organization [WHO], 2022). In Sudan, visual impairment represents the largest category of disabilities, accounting for an estimated 26–31% of the population with disabilities in a country of 44.91 million (as of 2021) (Ngubane and Zongozzi, 2021). Visual impairment affects learning by impacting cognitive integration and the processing of information (Al-Abbas, 2010). Cassar et al. (2022) evaluations of vision challenged children’s nonverbal intelligence discovered that this population may employ distinct verbal and working memory techniques for nonverbal tasks, necessitating the deployment of an adjusted instrument for assessment. Interest in adapting standardized assessments in Sudan dates back to 1946 (Scott, 1948), while Badri (1966) undertook a standardization of the Draw-a-Person Test in 1964; however, this is a psychological assessment rather than an intelligence test. Several studies related to intelligence testing have been conducted focusing specifically on the visually impaired population, including that of Al-Hussein and Muhammad (2014). Presently, the visually impaired in Sudan do not have a dedicated intelligence measure and the assessment currently in use universally is the instrument for the sighted.

Hayes, a pioneer in intelligence testing for the blind, asserted that only the verbal indices of intelligence scales can be used for the visually impaired—although this theory has been disputed (Morash and McKerracher, 2017). Notably, no measure of intelligence has been specifically adapted for visually impaired Sudanese children, with the verbal component of the WISC being applied without adaptation or validation (Al-Hussein and Muhammad, 2014).

Rindermann et al. (2020) revealed that visually impaired individuals tend to outperform their sighted peers on the working memory index but score lower on the verbal comprehension index. This inadequacy hinders educational access and achievement for the visually impaired, as intelligence testing often determines school placement.

To address these gaps, Minks et al. (2020) suggested augmenting intelligence assessments of visually impaired students with qualitative insights from teachers and parents. Such an approach offers a more comprehensive understanding when fully adapted instruments are unavailable.

## Intelligence and other assessments of the visually impaired

Numerous research have established new or modified tools for use with the target audience. Chen et al. (2021) examined the reliability and validity of the VCI (*Verbal Comprehension Index*) and WMI *(Working Memory Index)* of the WISC-IV with blind children in China.

Similarly, Ballesteros et al. (2005) developed a psychological test battery using a tactile adaptation of 20 subtests and tested it on an experimental group of 59 visually impaired students and a control group of 60 sighted students in Spain. Although insightful, the urban-focused study has limited generalizability, particularly to Sudan, given the vast cultural, linguistic, and geographical differences between Spain and Arab countries, including Sudan.

Raven’s Colored Progressive Matrices (CPM) was developed by John Raven (1902–1970) in the late 1930s to measure non-verbal intelligence, a globally recognized assessment. Rich and Anderson in 1965 developed a tactual adaptation of the CPM and tested it on a group of adults with “99% loss of full visual acuity” comprising 79 males and 43 females (Anderson, as cited in Curtis, 1972).

While this study highlights the importance of adapting intelligence assessments for the visually impaired, it focused on adults, did not use the WISC-IV, and was conducted in the United States, making it less relevant to the current study. This test has only limited applicability, though, due to its age and other factors.

## Modern tests for assess intelligence for individuals with visual impairments

In addition to verbal measures, researchers have developed haptic intelligence tests by adapting traditional performance subtests into tactile formats. Tactile versions of Raven’s Progressive Matrices and the Test of Nonverbal Intelligence (TONI) have been designed using raised-line or 3D materials, enabling individuals with visual impairments to engage in nonverbal reasoning tasks through touch. These adaptations have demonstrated validity in evaluating cognitive abilities beyond verbal skills (Armstrong, 2002; Layton and Koenig, 1998).

These methods reflect the broader need for adapted intelligence assessments that accommodate the sensory modalities of individuals with visual impairments, ensuring that their cognitive potential is measured as accurately and equitably as possible.

### Research in the Arab world

In our survey of the literature, we found little relevant research conducted in the Arab world overall. Among these was Dahir’s (2018) adaptation of the verbal comprehension and working memory indices of the WISC-IV for use with blind children in Palestine, which involved a study sample of 180 children (98 males and 82 females). However, this study has limited applicability to the present context. Another study by Abdel-Fattah (2010) developed a 60-item intelligence test for blind students in primary and secondary schools in Egypt focusing on linguistic and arithmetic skills. The test demonstrated acceptable reliability with Cronbach’s alpha (0.805), split-half test (0.781), and Guttmann’s reliability of 0.77. While these studies might have more relevance to Sudan, since the two countries share a border and important demographic characteristics, they did not utilize the WISC-IV. Sudan is characterized by great cultural diversity that is impacted by the traits of both the Arab and African (Nubian) background of its people.

### Purpose of the study

Therefore, there is currently no tool specifically designed or adapted to suit the needs of visually impaired Sudanese children for placement in accommodation and other educational services, such as counseling. Few studies have been conducted to create thorough and easily accessible intelligence evaluations for blind and visually impaired children in the Arab world, and specifically none have been found for Sudan.

### Aims

The primary goal of this study was to adapt and validate selected components of the Wechsler Intelligence Scale for Children—Fourth Edition (WISC-IV) for use with visually impaired children in Sudan. Specifically, the study focused on the Verbal Comprehension Index (VCI) and the Working Memory Index (WMI), as these rely on auditory and verbal processing rather than visual input. The adaptation aimed to ensure cultural, linguistic, and accessibility relevance for Sudanese students. By evaluating the reliability and validity of these adapted subtests, the study sought to develop a standardized, accessible tool for assessing the cognitive abilities of visually impaired children, enabling informed decisions about educational placement, support services, and psychological counseling.

## Materials and methods

### Participants

The research population included all visually impaired school students in Khartoum, totaling 179 students (106 males and 73 females). Totally blind students, in basic and secondary public and private schools, aged 6–16 years, with no other disability. Researchers obtained ethical approval from the University of Khartoum and permission from the Khartoum State Ministry of Education to administer the scale. The participants were confirmed to have visual impairments requiring visual aids but had no additional disabilities.

The scale was applied individually with the help of research assistants, primarily during the students’ morning class period. After excluding incomplete test forms, the final study sample consisted of 166 students (96 males and 70 females. The age distribution of the final sample was as follows: 6 years (3/1.80%), 7 years (10/6.02%), 8 years (15/9.03%), 9 years (21/12.65%), 10 years (11/6.62%), and 11 years (11/6.62%), 12 years (25/15.06%), 13 years (19/11.44%), 14 years (19/11.44%), 15 years (11/6.62%), and 16 years (21/12.65%).

### Instrument

The Wechsler Intelligence Scale for Children—Fourth Edition (WISC-IV) is a standardized, individually administered intelligence test designed to assess the cognitive abilities of children aged 6–16 years. Developed by David Wechsler, the WISC-IV evaluates a child’s intellectual functioning across four main indices:

   1.  Verbal Comprehension Index (VCI)—measures verbal reasoning, concept formation, and acquired knowledge.
   2.  Perceptual Reasoning Index (PRI)—assesses non-verbal and fluid reasoning, visual-spatial processing, and problem-solving.
   3.  Working Memory Index (WMI)—evaluates the ability to temporarily hold and manipulate information.
   4.  Processing Speed Index (PSI)—measures speed and accuracy of visual identification, decision-making, and graphomotor tasks.

The WISC-IV provides a Full-Scale IQ (FSIQ) score, which represents overall cognitive ability, along with scores for each index to identify strengths and weaknesses in specific cognitive domains. It is widely used in educational, clinical, and psychological settings for diagnosis, placement, and intervention planning (Wechsler, 2003).

The WISC-IV standardized for Arabic speakers in Egypt (Al-Buhairi, 2017a) was adapted for this study. The focus was on the verbal section, deemed suitable for visually impaired children. The test consisted of eight subtests categorized under two indices:

1. Verbal Comprehension Index

   1.  Similarities: A primary test consisting of 23 questions. Participants identify similarities between two familiar items or concepts.
   2.  Vocabulary: A primary test consisting of 36 questions (four converted into three-dimensional representations) measuring word knowledge.
   3.  Comprehension: A primary test consisting of 21 questions assessing understanding of social norms and practical knowledge.
   4.  Information: A secondary test consisting of 33 questions evaluating general knowledge.
   5.  Word Inference: A secondary test consisting of 24 questions (including one added for adaptation), requiring recognition of a concept from descriptive hints.

2. Working Memory Index

   1.  Digit Span: A primary test consisting of 16 questions involving forward, reverse, and ascending recall of number sequences by children.
   2.  Letter-Number Sequencing: (Primary test, 10 questions) requiring reorganization of mixed letters and numbers.
   3.  Arithmetic: A secondary test consisting of 34 questions, the first five of which were converted into tactile forms. Participants solve timed arithmetic problems mentally. This is the only timed test.
   4.  The test modifications addressed the specific needs of visually impaired students, ensuring accessibility through tactile and auditory adaptations.

### Translation

Al-Buhairi (2017a) used back-translation and consulted experts in assessment and psychological measurement with a background in translation of specific psychological terms. A wide range of information sources were utilized to formulate the scale, including specialists in the Arabic language whose insights were utilized to make the scale clearer and more concise. Al-Buhairi’s (2017a) approach to adjusting the scale for Arabic aligns with the method previously implemented by Al-Hussein (2005, 2008) in making the WISC-III suitable for Khartoum and other areas in the country. In preparation for the pilot study, a number of items were modified to suit the Sudanese Arabic dialect Arabic for our research. Due to financial constraints in developing countries, researchers resorted to using the Egyptian version (Al-Buhairi, 2017b), instead of the latest version. This version is easily accessible and was used with sighted individuals, so there was no need to discuss its psychometric characteristics.

### Face validity

In adapting the WISC-IV for visually impaired children, Al-Buhairi (2017a) excluded the Fluid Reasoning and the Processing Speed indices, which require vision, from the Arabic translation of the full test. The answer form, implementation guide, and correction procedures were also reformulated. To confirm the suitability of converted tactual items, the Word Inference Test and picture questions were tested with nine students aged 7–13 years at the Al-Noor Institute for the blind in Khartoum. No issues were found in understanding or answering the questions.

A total of 46 out of 198 questions were modified, including 9 tactual and 37 verbal questions, representing 23.23% of the scale. The total number of questions increased from 197 to 198 after adding one item to the Word Inference Test, as suggested by an expert from the Al-Noor Institute. Modifications included:

   a.  25.36% of the Verbal Comprehension Index and 17.67% of the entire scale.
   b.  Items modified across various tests: Similarities (1 item), Vocabulary (10 items, 4 tactual), Comprehension (8 items), Information (12 items), Word Inference (4 items, 1 added), and Arithmetic (11 items, 5 tactual).
   c.  No changes were made to the Digit Span or Letter-Number Sequencing tests.

The modified scale was approved by a group of nine experts from the Al-Noor Institute, including university professors and psychologists.

In many items, words that infer sightedness were omitted even if they could be understood, e.g., Item 1 of the Information Test is phrased: “Show me your foot.” This was replaced by, “(Where is) your foot?” to avoid a potentially negative psychological effect on the examinees, as well as to enhance the study’s findings and prevent errors in the validity of the scale. Moreover, we included the characteristics of the visually impaired in the implementation and correction guide to serve as an easily accessible reference for examiners, because knowledge of these characteristics is important to the successful delivery of the test. Therefore, our adapted tool comprised of 198 items, 138 for the VCI (*Verbal Comprehension Index*) and 60 for the WMI *(Working Memory Index).*

### Examiners

In the study, we recruited graduate psychology students from three Sudanese universities- Khartoum University, Al-Neelain University, and Omdurman Islamic University- were recruited to assist in administering the intelligence scale. The participants had to have at least a bachelor’s degree and applicants were asked about their experience with intelligence measures, and working with visually impaired individuals. A total of 80 people volunteered to participate, 13 of whom were trained. Of these trained volunteers, six examiners were selected.

The training process involved individualized instruction on the scale, its implementation, correction guide, and answer form. The examiners practiced administering the scale to children from the pilot group and scored the assessments under the supervision of the research team. They also observed the research team administering the scale. The training included discussions on working with visually impaired students. After this comprehensive process, the research team was confident in the examiners’ ability to reliably administer the scale.

### Statistical methods

To ensure the validity and reliability of the instrument, several statistical procedures were employed. Measures of central tendency and dispersion, including the mean, median, mode, standard deviation, and skewness, were calculated. Furthermore, item analysis was conducted to determine the difficulty and easiness indices as well as the discrimination power of the test items.

Internal consistency validity was examined by computing the correlation coefficients between each subscale, the other subscales, and the total score. Discriminant validity was evaluated by comparing the responses across different chronological age groups using one-way ANOVA followed by Tukey’s *post-hoc* test to detect significant differences.

To assess the reliability of the instrument, multiple methods were applied, including Cronbach’s alpha, the split-half method using the Spearman-Brown coefficient, and the test-retest method.

## Results

Tables 1–19 show the results of the study. Tables 1–5 focus on appropriateness of the two indices for the study population; Tables 6–18 address the validity of these indices; while Table 19 shows their reliability.

**TABLE 1:** Statistical indices of performance in the sub-tests.

Index	*M*	Mdn	Mode	SD	Skewness
Similarities	12.82	12	11	5.55	0.09
Digit span	20.62	22	23	6.36	−0.77
Vocabulary	15.89	15	14	5.49	0.46
Letter sequencing	23.53	25	26	10.73	−0.36
Comprehension	13.24	13	9	5.82	0.09
Information	33.54	35	17	18.10	0.06
Arithmetic	14.87	14	14	4.52	0.64
Word inference	17.17	15.5	0	11.28	0.13

**TABLE 2:** Difficulty coefficients of the VCI.

Item	Diff. coef.	Item	Diff. coef.	Item	Diff. coef.	Item	Diff. coef.	Item	Diff. coef.
**Similarities**									
1	0.78	6	0.58	11	0.32	16	0.27	21	0.22
2	0.76	7	0.55	12	0.51	17	0.33	22	0.23
3	0.72	8	0.51	13	0.44	18	0.26	23	0.21
4	0.73	9	0.54	14	0.39	19	0.34		
5	0.63	10	0.46	15	0.38	20	0.23		
**Vocabulary**									
1	0.98	9	0.82	17	0.40	25	0.48	33	0.38
2	1.00	10	0.69	18	0.23	26	0.38	34	0.38
3	1.00	11	0.52	19	0.52	27	0.48	35	0.47
4	0.92	12	0.48	20	0.47	28	0.22	36	0.46
5	0.88	13	0.85	21	0.29	29	0.41		
6	0.89	14	0.55	22	0.50	30	0.22		
7	0.60	15	0.42	23	0.42	31	0.44		
8	0.82	16	0.40	24	0.40	32	0.36		
**Comprehension**									
1	0.94	6	0.61	11	0.60	16	0.48	21	0.27
2	0.78	7	0.63	12	0.57	17	0.39		
3	0.67	8	0.67	13	0.46	18	0.35		
4	0.86	9	0.59	14	0.46	19	0.32		
5	0.86	10	0.65	15	0.32	20	0.34		
**Information**									
1	1.00	8	0.73	15	0.39	22	0.29	29	0.19
2	1.00	9	0.69	16	0.23	23	0.26	30	0.11
3	1.00	10	0.90	17	0.26	24	0.21	31	0.11
4	1.00	11	0.80	18	0.40	25	0.41	32	0.10
5	0.96	12	0.69	19	0.34	26	0.17	33	0.09
6	0.94	13	0.60	20	0.27	27	0.23		
7	0.96	14	0.52	21	0.26	28	0.22		
**Word inference**									
1	0.82	5	0.77	11	0.55	16	0.41	21	0.33
2	0.76	7	0.75	12	0.61	17	0.33	22	0.27
3	0.90	8	0.59	13	0.44	18	0.33	23	0.16
4	0.86	8	0.69	14	0.25	19	0.23	24	0.14
5	0.89	10	0.75	15	0.19	20	0.34	25	0.21

**TABLE 3:** Difficulty coefficients of the WMI.

Item	Diff. coef.	Item	Diff. coef.	Item	Diff. coef.	Item	Diff. coef.
Repeating numbers in ascending order				Repeating numbers in reverse order			
1	1.00	5	0.44	9	0.97	13	0.25
2	0.96	6	0.28	10	0.92	14	0.08
3	0.92	7	0.13	11	0.75	15	0.04
4	0.77	8	0.08	12	0.36	16	0.03
**Letter-number sequencing**							
1	0.97	4	0.75	7	0.34	10	0.18
2	0.96	5	0.58	8	0.20		
3	0.83	6	0.44	9	0.19		
**Arithmetic**							
1	0.98	10	0.84	19	0.69	28	0.22
2	0.98	11	0.83	20	0.52	29	0.16
3	0.97	12	0.80	21	0.44	30	0.11
4	0.97	13	0.73	22	0.39	31	0.05
5	0.92	14	0.70	23	0.54	32	0.04
6	0.94	15	0.70	24	0.51	33	0.05
7	0.91	16	0.65	25	0.44	34	0.03
8	0.91	17	0.69	26	0.35		
9	0.86	18	0.64	27	0.20		

**TABLE 4:** Discrimination coefficients of the VCI.

Item	Dis. coef.	Item	Dis. coef.	Item	Dis. coef.	Item	Dis. coef.	Item	Dis. coef.
**Similarities**									
1	0.42	6	0.64	11	0.59	16	0.54	21	0.38
2	0.47	7	0.83	12	0.78	17	0.66	22	0.47
3	0.54	8	0.88	13	0.88	18	0.52	23	0.42
4	0.52	9	0.90	14	0.78	19	0.69		
5	0.64	10	0.92	15	0.76	20	0.47		
**Vocabulary**									
1	0.02	9	0.26	17	0.76	25	0.97	33	0.76
2	0.09	10	0.57	18	0.47	26	0.76	34	0.76
3	0.09	11	0.85	19	0.95	27	0.97	35	0.95
4	0.16	12	0.54	20	0.90	28	0.45	36	0.92
5	0.23	13	0.28	21	0.59	29	0.83		
6	0.21	14	0.78	22	1.00	30	0.45		
7	0.78	15	0.85	23	0.85	31	0.88		
8	0.35	16	0.76	24	0.80	32	0.73		
**Comprehension**									
1	0.11	6	0.66	11	0.73	16	0.92	21	0.50
2	0.42	7	0.50	12	0.85	17	0.78		
3	0.64	8	0.54	13	0.83	18	0.66		
4	0.26	9	0.80	14	0.83	19	0.59		
5	0.26	10	0.69	15	0.64	20	0.64		
**Information**									
1	0.00	8	0.52	15	0.73	22	0.59	29	0.38
2	0.00	9	0.61	16	0.42	23	0.52	30	0.23
3	0.00	10	0.19	17	0.52	24	0.42	31	0.23
4	0.00	11	0.38	18	0.76	25	0.83	32	0.11
5	0.07	12	0.61	19	0.69	26	0.35	33	0.04
6	0.11	13	0.73	20	0.54	27	0.47		
7	0.11	14	0.90	21	0.52	28	0.11		
**Word inference**									
1	0.35	6	0.45	11	0.83	16	0.83	21	0.57
2	0.47	7	0.45	12	0.61	17	0.66	22	0.50
3	0.19	8	0.66	13	0.78	18	0.59	23	0.23
4	0.26	9	0.61	14	0.45	19	0.42	24	0.28
5	0.21	10	0.40	15	0.38	20	0.64	25	0.42

**TABLE 5:** Discrimination coefficients of the WMI.

Item	Diff. coef.	Item	Diff. coef.	Item	Diff. coef.	Item	Diff. coef.
Repeating numbers in ascending order				Repeating numbers in reverse order			
1	0.00	5	0.59	9	0.04	13	0.45
2	0.07	6	0.57	10	0.14	14	0.16
3	0.14	7	0.26	11	0.50	15	0.09
4	0.40	8	0.11	12	0.54	16	0.02
**Letter-number sequencing**							
1	0.04	4	0.40	7	0.59	10	0.04
2	0.07	5	0.73	8	0.35		
3	0.33	6	0.73	9	0.07		
**Arithmetic**							
1	0.02	10	0.30	19	0.57	28	0.45
2	0.02	11	0.33	20	0.85	29	0.33
3	0.04	12	0.38	21	0.83	30	0.23
4	0.04	13	0.52	22	0.73	31	0.11
5	0.14	14	0.54	23	0.85	32	0.09
6	0.11	15	0.59	24	0.97	33	0.11
7	0.16	16	0.64	25	0.78	34	0.07
8	0.16	17	0.61	26	0.71		
9	0.26	18	0.66	27	0.40		

**TABLE 6:** Correlations among subtests and the total score.

Sub-scale	Similari-ties	Digit span	Vocabulary	Letter-number sequencing	Comprehen-sion	Information	Arithmetic	Word inference	Index
Similarities	1.00								
Digit span	0.62**	1.00							
Vocabulary	0.81**	0.54**	1.00						
Letter-number sequencing	0.55**	0.55**	0.51**	1.00					
Comprehen-sion	0.80**	0.57**	0.84**	0.55**	1.00				
Information	0.80**	0.58**	0.72**	0.59**	0.76**	1.00			
Arithmetic	0.71**	0.63**	0.71**	0.60**	0.64**	0.80**	1.00		
Word inference	0.70**	0.35**	0.76**	0.57**	0.64**	0.77**	0.74**	1.00	
Total	0.91**	0.68**	0.92**	0.69**	0.89**	0.88**	0.84**	0.82**	1.00

*p < 0.05;

**p < 0.01.

**TABLE 7:** Correlation coefficients of the similarities test items.

Item	Corr. w/index	Corr. w/total score	Item	Corr. w/index	Corr. w/total score
1	0.66**	0.62**	13	0.70**	0.63**
2	0.64**	0.61**	14	0.74**	0.68**
3	0.65**	0.61**	15	0.69**	0.62**
4	0.64**	0.59**	16	0.68**	0.58**
5	0.65**	0.61**	17	0.60**	0.49**
6	0.53**	0.47**	18	0.61**	0.52**
7	0.81**	0.78**	19	0.62**	0.59**
8	0.83**	0.73**	20	0.50**	0.44**
9	0.82**	0.77**	21	0.39**	0.44**
10	0.76**	0.65**	22	0.37**	0.41**
11	0.65**	0.51**	23	0.42**	0.39**
12	0.69**	0.66**			

*p < 0.05;

**p < 0.01.

**TABLE 8:** Correlation coefficients of the VCI.

Item	Corr. w/index	Corr. w/total score	Item	Corr. w/index	Corr. w/total score
1	0.17	0.20	19	0.91**	0.84**
2	0.29*	0.30*	20	0.68**	0.61**
3	0.28*	0.32*	21	0.60**	0.55**
4	0.38**	0.39**	22	0.89**	0.84**
5	0.51**	0.46**	23	0.70**	0.69**
6	0.45**	0.37**	24	0.59**	0.49**
7	0.68**	0.69**	25	0.86**	0.78**
8	0.53**	0.53**	26	0.68**	0.54**
9	0.58**	0.50**	27	0.74**	0.75**
10	0.71**	0.64**	28	0.43**	0.46**
11	0.74**	0.76**	29	0.61**	0.61**
12	0.61**	0.51**	30	0.29*	0.36**
13	0.55**	0.52**	31	0.77**	0.64**
14	0.80**	0.79**	32	0.69**	0.60**
15	0.52**	0.57**	33	0.55**	0.52**
16	0.40**	0.43**	34	0.44**	0.32*
17	0.68**	0.63**	35	0.79**	0.67**
18	0.48**	0.52**	36	0.85**	0.71**

*p < 0.05;

**p < 0.01.

**TABLE 9:** Correlation coefficients of the comprehension test items.

Item	Corr. w/index	Corr. w/total score	Item	Corr. w/index	Corr. w/total score
1	0.49**	0.34**	12	0.83**	0.70**
2	0.68**	0.57**	13	0.67**	0.58**
3	0.69**	0.66**	14	0.71**	0.63**
4	0.66**	0.50**	15	0.37**	0.33**
5	0.62**	0.46**	16	0.68**	0.75**
6	0.67**	0.64**	17	0.53**	0.53**
7	0.62**	0.41**	18	0.55**	0.52**
8	0.69**	0.59**	19	0.62**	0.65**
9	0.76**	0.79**	20	0.53**	0.56**
10	0.78**	0.68**	21	0.50**	0.55**
11	0.71**	0.64**			

*p < 0.05;

**p < 0.01.

**TABLE 10:** Correlation coefficients of the information test items.

Item	Corr. w/ index	Corr. w/total score	Item	Corr. w/index	Corr. w/total score	Item	Corr. w/index	Corr. w/total score
1	0.00	0.00	12	0.65**	0.67**	23	0.64**	0.54**
2	0.00	0.00	13	0.61**	0.59**	24	0.53**	0.41**
3	0.00	0.00	14	0.76**	0.85**	25	0.76**	0.69**
4	0.00	0.00	15	0.40**	0.40**	26	0.52**	0.42**
5	0.16	0.14	16	0.55**	0.46**	27	0.58**	0.43**
6	0.36**	0.33**	17	0.51**	0.43**	28	0.46**	0.33*
7	0.21	0.18	18	0.76**	0.66**	29	0.56**	0.45**
8	0.66**	0.61**	19	0.57**	0.51**	30	0.57**	0.37**
9	0.69**	0.66**	20	0.49**	0.40**	31	0.57**	0.37**
10	0.45**	0.45**	21	0.56**	0.43**	32	0.53**	0.32*
11	0.53**	0.48**	22	0.63**	0.51**	33	0.49**	0.26*

*p < 0.05;

**p < 0.01.

**TABLE 11:** Correlation coefficients of the word inference test items.

Item	Corr. w/index	Corr. w/total score	Item	Corr. w/index	Corr. w/total score
1	0.42**	0.43**	14	0.33**	0.20
2	0.52**	0.50**	15	0.59**	0.40**
3	0.43**	0.37**	16	0.70**	0.60**
4	0.55**	0.45**	17	0.58**	0.45**
5	0.54**	0.38**	18	0.69**	0.54**
6	0.50**	0.45**	19	0.53**	0.35**
7	0.52**	0.47**	20	0.68**	0.55**
8	0.58**	0.47**	21	0.59**	0.40**
9	0.68**	0.66**	22	0.55**	0.39**
10	0.57**	0.34**	23	0.50**	0.44**
11	0.64**	0.55**	24	0.46**	0.28**
12	0.67**	0.57**	25	0.48**	0.40**
13	0.59**	0.58**			

*p < 0.05;

**p < 0.01.

**TABLE 12:** Correlation coefficients of the digit span test items.

Item	Corr. w/index	Corr. w/total score	Item	Corr. w/index	Corr. w/total score
1	0.67**	0.42**	9	0.33**	0.25*
2	0.51**	0.27**	10	0.46**	0.35**
3	0.54**	0.32**	11	0.67**	0.54**
4	0.57**	0.40**	12	0.71**	0.59**
5	0.59**	0.29*	13	0.71**	0.45**
6	0.61**	0.45**	14	0.59**	0.36**
7	0.37**	0.23	15	0.36**	0.18
8	0.84**	0.51**	16	0.34**	0.12

*p < 0.05;

**p < 0.01.

**TABLE 13:** Correlation coefficients of the digit span test items.

Item	Corr. with index	Corr. with total score
1	0.30*	0.20
2	0.43**	0.27*
3	0.81**	0.57**
4	0.90**	0.60**
5	0.84**	0.54**
6	0.87**	0.66**
7	0.74**	0.48**
8	0.58**	0.38**
9	0.43**	0.32*
10	0.41**	0.33*

*p < 0.05;

**p < 0.01.

**TABLE 14:** Correlation coefficients of the arithmetic test items.

Item	Corr. w/index	Corr. w/total score	Item	Corr. w/index	Corr. w/total score
1	0.33**	0.11	18	0.71**	0.62**
2	0.33**	0.11	19	0.75**	0.59**
3	0.43**	0.23	20	0.66**	0.66**
4	0.42**	0.20	21	0.71**	0.78**
5	0.58**	0.37**	22	0.65**	0.66**
6	0.47**	0.28*	23	0.75**	0.63**
7	0.63**	0.42**	24	0.76**	0.77**
8	0.48**	0.32*	25	0.69**	0.68**
9	0.55**	0.38**	26	0.60**	0.63**
10	0.71**	0.49**	27	0.42**	0.37**
11	0.56**	0.47**	28	0.44**	0.49**
12	0.71**	0.53**	29	0.37**	0.40**
13	0.71**	0.53**	30	0.33**	0.34**
14	0.73**	0.62**	31	0.27	0.27
15	0.75**	0.54**	32	0.22	0.18
16	0.67**	0.57**	33	0.30*	0.27*
17	0.75**	0.58**	34	0.28	0.18

*p < 0.05;

**p < 0.01.

**TABLE 15:** One-way ANOVA for the differences in intelligence by age.

Test	Source of variance	Sum of squares	*df*	Mean squares	*F*	Sig.
Verbal comprehension	Between groups	2,19,413.28	3	73,137.76	80.01	0.00
	Within groups	1,48,085.66	162	914.10		
	Total	3,67,498.94	165			
Working memory	Between groups	17,843.57	3	5,947.85	57.05	0.00
	Within groups	16,889.29	162	104.25		
	Total	34,732.87	165			
Total scale	Between groups	3,61,282.95	3	1,20,427.65	90.70	0.00
	Within groups	2,14,997.37	162	1,327.14		
	Total	5,76,280.33	165			

**TABLE 16:** *Post-hoc* tests- multiple comparisons -Tukey HSD.

Dependent variable	(I) 4 groups	(J) 4 groups	Mean difference (I−J)	Std. error	Sig.	95% confidence interval	
						Lower bound	Upper bound
Verbal comprehension	1	2	−34.39618*	7.34201	0.000	−53.4553	−15.3371
		3	−69.87662*	7.30903	0.000	−88.8501	−50.9031
		4	−101.18277*	7.11129	0.000	−119.6430	−82.7226
	2	1	34.39618*	7.34201	0.000	15.3371	53.4553
		3	−35.48044*	6.48333	0.000	−52.3105	−18.6504
		4	−66.78659*	6.25956	0.000	−83.0358	−50.5374
	3	1	69.87662*	7.30903	0.000	50.9031	88.8501
		2	35.48044*	6.48333	0.000	18.6504	52.3105
		4	−31.30615*	6.22084	0.000	−47.4548	−15.1575
	4	1	101.18277*	7.11129	0.000	82.7226	119.6430
		2	66.78659*	6.25956	0.000	50.5374	83.0358
		3	31.30615*	6.22084	0.000	15.1575	47.4548
Working Memory	1	2	−12.14950*	2.47950	0.000	−18.5860	−5.7130
		3	−23.13312*	2.46836	0.000	−29.5407	−16.7255
		4	−28.97759*	2.40158	0.000	−35.2119	−22.7433
	2	1	12.14950*	2.47950	0.000	5.7130	18.5860
		3	−10.98362*	2.18951	0.000	−16.6674	−5.2999
		4	−16.82809*	2.11394	0.000	−22.3157	−11.3405
	3	1	23.13312*	2.46836	0.000	16.7255	29.5407
		2	10.98362*	2.18951	0.000	5.2999	16.6674
		4	−5.84447*	2.10087	0.030	−11.2981	−0.3908
	4	1	28.97759*	2.40158	0.000	22.7433	35.2119
		2	16.82809*	2.11394	0.000	11.3405	22.3157
		3	5.84447*	2.10087	0.030	0.3908	11.2981
total	1	2	−46.54568*	8.84657	0.000	−69.5105	−23.5809
		3	−93.00974*	8.80684	0.000	−115.8714	−70.1481
		4	−130.16036*	8.56857	0.000	−152.4035	−107.9172
	2	1	46.54568*	8.84657	0.000	23.5809	69.5105
		3	−46.46406*	7.81193	0.000	−66.7430	−26.1851
		4	−83.61468*	7.54230	0.000	−103.1937	−64.0357
	3	1	93.00974*	8.80684	0.000	70.1481	115.8714
		2	46.46406*	7.81193	0.000	26.1851	66.7430
		4	−37.15062*	7.49565	0.000	−56.6086	−17.6927
	4	1	130.16036*	8.56857	0.000	107.9172	152.4035
		2	83.61468*	7.54230	0.000	64.0357	103.1937
		3	37.15062*	7.49565	0.000	17.6927	56.6086

*The mean difference is significant at the 0.05 level.

**TABLE 17:** Tukey’s *post-h*oc test for the differences in verbal comprehension by age.

Age groups (years)	Sub-groups according to significance of differences				
	*N*	1	2	3	4
6–8	28	44.4			
9–11	43		78.8		
12–13	44			114.3	
14–16	51				145.6

**TABLE 18:** Tukey’s *post-hoc* test for the differences in working memory by age.

Age groups (years)	Sub-groups according to significance of differences				
	*N*	1	2	3	4
6–8	28	30.5			
9–11	43		42.7		
12–13	44			53.7	
14–16	51				59.5

**TABLE 19:** Tukey’s *post-hoc* test for the differences in the total score by age.

Age groups (years)	Sub-groups according to significance of differences				
	*N*	1	2	3	4
6–8	28	75.03			
9–11	43		121.5		
12–13	44			168.04	
14–16	51				205.1

### Appropriateness of the two indices

#### Central tendency measures

The mean, median, and mode values in tests of digit span, letter sequencing, comprehension, information, arithmetic, and word inference were close to normal distribution (Table 1).

#### Item difficulty

Faraj (2007) suggests grading scale items based on difficulty, calculated by dividing the correct answers by sample size. Difficulty coefficients were calculated for each subtest and integrated. The difficulty coefficients were calculated by arranging the data in descending order, taking the highest and lowest quartiles of 42 items, i.e., 25.30%, and used the equation L+D 2⁢N (Abdul-Rahman, 2008), where (*L*) denotes the number of participants in the upper quartile, (*D*) denotes the number of participants in the lower quartile, and (2*N*) denotes the total number of individuals in the upper and lower quartiles. Table 2 shows the difficulty coefficients for the VCI.

The difficulty coefficients of the Verbal Comprehension Index ranged from 0.21 to 0.78 for similarities, 0.22–1.00 for vocabulary, 0.21–0.94 for comprehension, 0.09–1.00 for information, and 0.14–0.90 for word inference, while the Working Memory Index had difficulty coefficients ranging from 0.03 to 1.00 for digit span, 0.18–0.97 for letter-number sequencing, and 0.03–0.98 for arithmetic (Table 3).

The finding showed that 56.06% of the items had a difficulty level ranging from 25 to 75%, while 18.68% lower than 25%, and 25.25% with a difficulty level higher than 75%.

#### Item discrimination

The discrimination coefficient for each item was calculated using the equation $\frac{L-D}{N}$, where (*L*) denotes the number of participants in the top quartile who answered the item correctly, (*D*), the number of participants in the lower quartile, and (*N*), the number of participants in one of the two quartiles. Out of 166 participants, 42 were chosen, with a rate of 25.30% since they could not be divided into four groups equally. The discrimination coefficients were extracted for all subtests and the tests of all the indices integrated (Table 4).

The Verbal Comprehension Index coefficients ranged from 0.42 to 0.92 for similarities, 0.02–1.00 for vocabulary, 0.11–0.92 for comprehension, 0–0.90 for information, and 0.19–0.83 for word inference (Table 4). For the Working Memory Index, the discrimination coefficients ranged from 0 to 0.59 for digit span, 0.04–0.73 for letter-number sequencing, and 0.02–0.97 for arithmetic (Table 5).

The scale items had graded discrimination coefficients higher values generally accepted (Tables 4, 5).

### Validity of the two indices

#### Internal consistency of the verbal comprehension index

The scale’s internal consistency was determined by calculating correlation coefficients among dimensions and total score. The correlation coefficients among the subtests of the Verbal Comprehension Index ranged from 0.84 to 0.64, and the correlation coefficients between them and the total score ranged from 0.82 to 0.92. The correlation coefficients among the subtests of the Working Memory Index ranged from 0.51 to 0.63, and the correlation coefficients between them and the total score ranged from 0.68 to 0.84. The correlation coefficients between the tests in the two indices ranged from 0.35 to 0.80, all statistically significant (*p* ≤ 0.01) (Table 6).

##### Similarities test

The items of the similarities test correlated with the index with coefficients ranging from 0.37 to 0.83 and correlated with the total score with coefficients ranging from 0.39 to 0.78, all significant at *p* ≤ 0.01 (Table 7).

##### Vocabulary test

The vocabulary test items correlated with the index with coefficients ranging from 0.17 to 0.91 and correlated with the total score with coefficients ranging from 0.20 to 0.84, all significant at *p* ≤ 0.01, except for one item that was significant at *p* ≤ 0.5 (Table 8).

##### Comprehension test

Table 9 shows that the comprehension test items correlated with the index with coefficients ranging from 0.49 to 0.83 and total score with coefficients ranging from 0.33 to 0.79, all significant at *p* ≤ 0.01.

##### Information test

The information test items correlated with the index with coefficients ranging from 0.16 to 0.76 and with total score with coefficients ranging from 0.14 to 0.85, with *p* ≤ 0.05, except for Items 5 and 7 (Table 10).

##### Word inference test

The information test items correlated with the index with coefficients ranging from 0.33 to 0.70 and with the total score with coefficients ranging from 0.20 to 0.66, all statistically significant at *p* ≤ 0.01, except for Item 14 whose correlation with the total score is not significant (Table 11).

#### Internal consistency of the working memory index

##### Digit span test

The digit span test items correlated with the index with coefficients ranging from 0.33 to 0.84, and with the total score with coefficients ranging from 0.12 to 0.59, all significant at *p* ≤ 0.05, except for three items that did not correlate with the total score (Table 12).

##### Letter-number sequencing test

The letter-number sequencing test items correlated with the index with coefficients ranging from 0.30 to 0.90 and *t*-test with the total score with coefficients ranging from 0.20 to 0.66, all significant at *p* ≤ 0.05, except for one item that did not correlate with the total score (Table 13).

##### Arithmetic test

The arithmetic test items showed correlations with both the index and total score coefficients ranging from 0.22 to 0.76, and 0.11–0.78; all significant at *p* ≤ 0.05, except for three items not correlating with the index and seven items not correlating with the total score.

Tables 6–14 show that the scale items strongly correlated with the indices and the total score. Most correlations were significant at *p* ≤ 0.01 level, a few were significant at *p* ≤ 0.05 level, and very few—14 of the total items or only 7.07%—did not correlate either with the indices or the total score.

#### Discriminatory validity

The study aimed to establish the discriminatory validity of a scale by grouping participants based on age. Due to low sample sizes in certain age groups (e.g., only three 6-year-old participants), the participants were divided into four groups: 6–8 years (*n* = 28), 9–11 years (*n* = 43), 12–13 years (*n* = 44), and 14–16 years (*n* = 51). Then the discriminatory validity was examined using one-way ANOVA and Tukey’s *post-hoc* test (Tables 15, 16).

The *f*-values were 80.01, 57.05, and 90.7 for verbal comprehension, working memory, and total score, respectively (Table 15). The probabilistic value was significant (*p* ≤ 0.05). This indicates that there were differences in intelligence in the two indices and the total score by age. To find out the direction of the differences, Tukey’s *post-hoc* test was used. (Table 16) shows the *post-hoc* Tests, Multiple Comparisons of Tukey HSD test. The means of intelligence in the Verbal Comprehension Index were 44.4, 78.8, 114.3, and 145.6 for age groups 6–8, 9–11, 12–13, and 14–16, respectively (Table 17). The means of intelligence in the Working Memory Index were 30.5, 42.7, 53.7, and 59.5 for age groups 6–8, 9–11, 12–13, and 14–16, respectively (Table 18).

The means of intelligence in the total score were 75.03, 121.5, 168.04, and 205.1 for age groups 6–8, 9–11, 12–13, and 14–16, respectively (Table 19).

#### Reliability of the two indices

The scale’s reliability was evaluated using Cronbach’s alpha, split-half, and test-retest methods (Table 20). Subtest reliability coefficients ranged from 0.79 to 0.95 (Cronbach’s alpha), 0.60–0.87 (split-half), and 0.59–0.87 (test-retest). The VCI showed reliability coefficients of 0.98 (Cronbach’s alpha), 0.94 (split-half), and 0.91 (test-retest). The reliability coefficients of the working memory index were 0.93, 0.84, and 0.89 for Cronbach’s alpha, split-half, and test-retest, respectively. The total scale’s reliability coefficients were 0.98 (Cronbach’s alpha), 0.93 (split-half), and 0.94 (test-retest). Internal consistency was confirmed using Cronbach’s alpha, split-half, and re-testing.

**TABLE 20:** Reliability coefficients of the scale.

Sub-scale	Cronbach’s α	Split-half	Test-retest
Similarities	0.93	0.84	0.85
Digit span	0.79	0.67	0.82
Vocabulary	0.95	0.87	0.85
Letter-number sequencing	0.85	0.83	0.59
Comprehension	0.93	0.84	0.83
Information	0.91	0.68	0.85
Arithmetic	0.94	0.73	0.87
Word inference	0.90	0.60	0.76
VCI	0.98	0.94	0.91
WMI	0.93	0.84	0.89
Total scale	0.98	0.93	0.94

## Discussion

The results indicate that the participants’ scores approximated a normal distribution, as evidenced by the near equivalence of the mean, median, and mode (Al-Aker, 2015). Additionally, the relatively low skewness values—whether positive or negative—suggest a distribution that tends toward normality, which is essential for statistical analyses such as calculating discrimination coefficients. The difficulty coefficients were within acceptable limits, with 56.06% of the items falling within the 25–75% range. This is consistent with Abdul-Rahman’s (2008) recommendation that approximately half of the test items should fall within this range to be considered appropriately challenging. Likewise, Abu-Allam (2006) noted that acceptable difficulty levels generally range between 20 and 80%.

The scale also demonstrated satisfactory and graduated discrimination indices. According to Al-Khatib and Abd Al-Rahim (2010), a discrimination coefficient of 0.16 or higher is considered acceptable. Notably, approximately two-thirds of the items (128 items, or 64.64%) had discrimination coefficients exceeding 0.40, indicating strong discriminative power. Seven items recorded zero discrimination coefficients; this outcome is attributed to their placement before the test’s starting point for certain age groups (see Tables 4, 5). The use of age-based starting points ensures that examinees begin the test at a level appropriate for their developmental stage (Al-Buhairi, 2017b). For instance, a 12-year-old would begin at item 5, and correctly answering these items would result in automatic credit for preceding items, thus reducing test fatigue and maintaining engagement. Despite such procedural adjustments, the success rate on these starting point items remained high—around 95%—across age groups, further supporting the reliability of the scale’s difficulty and discrimination measures.

The scale also exhibited strong internal consistency, with correlation coefficients among sub-tests and between sub-tests and the total score ranging from 0.51 to 0.92. One exception was the relatively weak correlation (0.35) between the word inference subtest and the digit span test, which may be attributed to fatigue effects, as the word inference test appears later in the assessment—an observation consistent with Dahir (2018). In general, the current study yielded higher correlation coefficients than previous studies in Sudan, such as Al-Hussein (2008), who reported notably lower inter-test correlations. For example, in the present adaptation, the correlation between the similarities and matrix reasoning subtests was only 0.038 in earlier work.

The observed strong inter-correlations among subtests and with the total score align with Spearman’s two-factor theory of intelligence, which posits the existence of both general and specific cognitive abilities. These findings are also consistent with earlier research (Al-Hussein, 2005, 2008; Dahir, 2018; Khaleefa, 2011), confirming the theoretical coherence of the scale.

The Verbal Comprehension Index (VCI) showed stronger correlations than the Working Memory Index (WMI), which may reflect cultural factors or the fact that the VCI underwent more substantial cultural and linguistic adaptation than the WMI. Furthermore, the study found that older participants scored higher on verbal comprehension, working memory, and overall test performance, which aligns with earlier findings (Al-Hussein, 2005, 2008; Dahir, 2018; Khaleefa, 2011), suggesting that raw intelligence increases with age through adolescence and into early adulthood.

In summary, the pattern of correlation coefficients and discrimination values supports the validity of the scale. Furthermore, the reliability coefficients obtained were high and consistent with those reported in prior research (Al-Bashir, 2004; Al-Hussein, 2005, 2008; Dahir, 2018; Hammad, 2012), underscoring the overall psychometric robustness of the adapted tool.

## Implications

This study demonstrates the practical applicability of the Verbal Comprehension and Working Memory Indices in assessing the verbal intelligence of students with visual impairments in Sudan. It offers a validated model for adapting intelligence measures to suit the needs of visually impaired populations, addressing a key limitation of earlier research that relied on tools designed for sighted individuals (Al-Hussein and Muhammad, 2014).

With a culturally and developmentally appropriate tool now available, these results can inform national policy and practice, including the development of new admission criteria for universities or specialized programs for gifted students. The scale’s demonstrated ability to predict academic potential offers a promising foundation for future educational planning and equitable access to learning opportunities for students with visual impairments.

## Limitations

The study aimed to adapt an international intelligence for visually-impaired students in Sudan, focusing on the importance of these tests. The study was limited in sample size and couldn’t obtain contact information from the Ministry of Education and the National Union of the Blind, for all students with visual impairment in Khartoum. Future research should replicate the present study with a larger sample and cover states other than Khartoum State, so that results can be more generalizable.

The study also employed the WISC-IV, which is not the recent version due to financial limitations in developing countries. Furthermore, the study failed to cover other indices of intelligence in the test such as the Perceptual Reasoning Index and the Processing Speed Index. Therefore, we recommend that future research covers these two indices to obtain more comprehensive information on the visually impaired. However, we believe that the current scale form is very useful for researchers and practitioners and fulfills the professional and research purposes for which it was designed. Finally, there is a need to expand the scope of standardized measures of intelligence and this requires financial support from official authorities.

## Data Availability

The data that support the findings of this study are available from the corresponding author upon reasonable request.

## References

[B1] Abdel-Fattah F. (2010). *IQ test for the blind.* Egypt: Arab Renaissance House.

[B2] Abdul-Rahman S. (2008). *Psychometrics theory and practice* 5th Edn. Egypt: Arab Nile Authority.

[B3] Abu-Allam R. (2006). *Research methods in psychological and educational sciences* 5th Edn. India: Universities Publishing House.

[B4] Al-Abbas R. (2010). *An introduction to the psychology of people with special needs.* Khartoum: Khartoum University Publishing House.

[B5] Al-Aker M. (2015). *Standardization of the intelligence test for the age group (4-6) years for kindergarten children in the governorates of Gaza.* Unpublished master’s thesis, Al-Aqsa University: Sungai Petani.

[B6] Al-Bashir M. (2004). *Modification of the fourth version of the Stanford Binet Scale to suit the Sudanese environment.* Unpublished doctoral dissertation, University of Khartoum: Khartoum.

[B7] Al-Buhairi A. (2017a). *The Wechsler Children’s Intelligence Scale: A technical and interpretive guide [WISC-IV].* Egypt: Anglo-Egyptian Library

[B8] Al-Buhairi A. (2017b). *The Wechsler Intelligence Scale for Children: A guide to application and correction [WISC-IV].* Egypt: Anglo-Egyptian Library.

[B9] Al-Hussein A. (2005). *Adapting and standardization of the Wechsler Scale for Children’s Intelligence - Third Edition, Khartoum State.* Unpublished master’s thesis, Al-Neelain University: Khartoum.

[B10] Al-Hussein A. A. (2008). *Adaptation and standardization of the Wechsler Intelligence Scale for Children - Third Edition in Sudan (WISC-III).* Unpublished doctoral dissertation, Al-Neelain University: Khartoum.

[B11] Al-Hussein A. Muhammad S. (2014). The level of general intelligence and creativity among students with visual disabilities at Al-Neelain University - Republic of Sudan. *Int. J. Excell. Dev.* 5 169–182.

[B12] Al-Khatib M. Abd Al-Rahim N. (2010). Validity and reliability indices of John Raven’s progressive matrices test. *J. Sci. Technol.* 11 169–194.

[B13] Allam S. (2000). *Educational and psychological measurement and evaluation.* Beirut: Arab Thought House.

[B14] Armstrong R. J. (2002). Adapting intelligence tests for individuals who are blind or visually impaired. *Rehabil. Psychol.* 47 131–141. 10.1037/0090-5550.47.2.131

[B15] Badri M. (1966). *The psychology of children’s drawings - the test of draw a man and its applications on Arab countries.* Sharjah: Dar Al-Fath.

[B16] Bahadır S. Bahadır O. (2024). Information of difficulties faced by visually impaired students in the learning process at school. *Trends Comp. Sci. Inform. Technol.* 9 81–89. 10.17352/tcsit.000085

[B17] Ballesteros S. Bardisa D. Millar S. Reales J. M. (2005). The haptic test battery: A new instrument to test tactual abilities in blind and visually impaired and sighted children. *Br. J. Vis. Impairment* 23 11–24. 10.1177/0264619605051717

[B18] Cassar C. Lucchese F. (2016). Psychometric test for blind adults and children, critical issues and perspectives. *Int. J. Dev. Educ. Psychol. Rev. INFAD Psicol.* 2 109–116. 10.17060/ijodaep.2016.n1.v2.150

[B19] Cassar C. Tambelli R. Pezzuti L. Lecis D. Castorina S. Ricci D. (2022). A haptic nonverbal cognitive test for children and adolescents with visual impairment. *J. Vis. Impairment Blindness* 116 485–495. 10.1177/0145482X221117172

[B20] Chen X. Lu M. Bu W. Wang L. Wang Y. Xu Y. (2021). Psychometric properties of WISC-IV verbal scales: a study of students in China who are blind. *J. Vis. Impairment Blindness* 115 228–241. 10.1177/0145482X211018520

[B21] Curtis W. S. (1972). *Development and application of intelligence tests for the blind: A research utilization conference.* Final report [Research Grant No. 22-P-55152/4-01]. New Delhi: Division of Research and Demonstration Grants, Social and Rehabilitation Service.

[B22] Dahir A. (2018). *Standardization of the Wechsler Intelligence Scale (indicators of verbal comprehension and working memory) on blind children in the Palestinian environment.* Unpublished master’s thesis, The Islamic University: India.

[B23] Faraj S. (2007). *Psychometrics* 6th Edn. Egypt: Anglo-Egyptian Library.

[B24] Hammad I. (2012). *Standardization of the colored progressive matrices test in the Palestinian environment.* Unpublished master’s thesis, The Islamic University: India.

[B25] Khaleefa O. (2011). Progressive matrices test norms in Khartoum state. *J. Psychol. Stud*. 5, 41–76.

[B26] Layton C. A. Koenig A. J. (1998). Tactile adaptations of intelligence tests. *J. Vis. Impairment Blindness* 92 328–334. 10.1177/0145482X9809200506

[B27] Minks A. Williams H. Basille K. (2020). A critique of the use of psychometric assessments with visually impaired children and young people. *Educ. Psychol. Pract.* 36 170–192. 10.1080/02667363.2020.1724894

[B28] Morash V. S. McKerracher A. (2017). Beware of intelligence resultsbased on common verbal tests. *J. Vis. Impairment Blindness* 111 187–190. 10.1177/0145482X1711100212

[B29] Ngubane S. A. Zongozzi J. N. (2021). Country report: Sudan. *Afr. Disabil. Rights Yearb*. 9, 254–272. 10.29053/2413-7138/2021/v9a12

[B30] Rindermann H. Ackermann A. L. Te Nijenhuis J. (2020). Does blindness boost working memory? A natural experiment and cross-cultural study. *Front. Psychol.* 11:1571. 10.3389/fpsyg.2020.01571 32719644 PMC7347789

[B31] Scott G. (1948). Intelligence testing in the Sudan. *Sudan Notes Rec.* 29 107–119.

[B32] Wechsler D. (2003). *Wechsler intelligence scale for children – Fourth Edition (WISC-IV).* San Antonio, TX: The Psychological Corporation.

[B33] World Health Organization [WHO] (2022). *Fact sheet: Blindness and vision impairment.* Geneva: World Health Organization.

